# Network pharmacology and gut microbiota insights: unraveling Shenling Baizhu powder’s role in psoriasis treatment

**DOI:** 10.3389/fphar.2024.1362161

**Published:** 2024-02-14

**Authors:** Bin Tang, Xuwei Zheng, Qianqian Luo, Xiong Li, Yujie Yang, Yang Bi, Yonggen Chen, Ling Han, Haiming Chen, Chuanjian Lu

**Affiliations:** ^1^ The Second Clinical College of Guangzhou University of Chinese Medicine, Guangzhou, China; ^2^ State Key Laboratory of Dampness Syndrome of Chinese Medicine, Guangdong Provincial Hospital of Chinese Medicine, The Second Affiliated Hospital of Guangzhou University of Chinese Medicine, Guangzhou, China; ^3^ Guangdong Provincial Clinical Medicine Research Center for Chinese Medicine Dermatology, Guangzhou, China; ^4^ Guangdong-Hong Kong-Macau Joint Lab on Chinese Medicine and Immune Disease Research, Guangzhou University of Chinese Medicine, Guangzhou, China

**Keywords:** Shenling baizhu powder, psoriasis, network pharmacology, gut microbiota, lipid metabolism

## Abstract

**Background:** Psoriasis, a chronic skin condition characterized by systemic inflammation and altered gut microbiota, has been a target of Traditional Chinese Medicine (TCM) for centuries. Shenling Baizhu Powder (SLBZP), a TCM formulation, holds promise for treating inflammatory diseases, but its specific role in psoriasis and impact on gut microbiota is not fully understood.

**Objective:** This study aims to elucidate the mechanism of SLBZP in treating psoriasis, integrating component analysis, network pharmacology, and experimental validation in mice models.

**Methods:** We commenced with a detailed component analysis of SLBZP using liquid chromatograph and mass spectrometer (LC-MS). Network pharmacology analysis was used to predict the potential action targets and pathways of SLBZP in psoriasis. An *in vivo* experiment was conducted with psoriasis mice models, treated with SLBZP. Therapeutic effects were assessed via symptomatology, histopathology, and immunohistochemical analysis. Gut microbiota composition was analyzed using 16S rRNA gene sequencing.

**Results:** A total of 42 main components and quality markers were identified, primarily from licorice and ginseng, including flavonoids, saponins and other markers. PPI topology analysis showed that TNF, IL-6, IL-1β, TP53 and JUN were the core DEPs. 168 signaling pathways including lipid and atherosclerosis, AGE-RAGE signaling pathway, IL-17 signaling pathway and Th17 cell differentiation were enriched by KEGG. SLBZP demonstrated significant therapeutic effects on psoriasis in mice, with alterations in skin pathology and biomarkers. Additionally, notable changes in gut microbiota composition were observed post-treatment, indicating a possible gut-skin axis involvement.

**Conclusion:** This research has pinpointed lipid metabolism as a key pathway in the treatment of psoriasis with SLBZP. It explores how SLBZP’s modulation of gut microbiota and lipid metabolism can alleviate psoriasis, suggesting that balancing gut microbiota may reduce inflammation mediators and offer therapeutic benefits. This underscores lipid metabolism modulation as a potential new strategy in psoriasis treatment.

## 1 Introduction

Psoriasis is a chronic, immune-mediated dermatological disorder, characterized by hyperproliferation of keratinocytes and extensive inflammation (Griffiths et al., 2021). This disease manifests as raised, red, scaly patches on the skin and is often associated with significant physical discomfort and psychosocial morbidity. The pathogenesis of psoriasis is complex and multifactorial, involving genetic predisposition, immunological dysregulation, and environmental triggers. Recent advances in understanding the pathophysiology of psoriasis have highlighted the role of T-helper cells, particularly Th17 cells (Guo et al., 2023; Lee et al., 2023), in driving the inflammatory cascade that characterizes this disease.

The gut-skin axis, a bidirectional communication pathway between the gastrointestinal tract and the skin, has emerged as a crucial player in the pathogenesis of various dermatological conditions, including psoriasis (Mahmud et al., 2022; Aguwa et al., 2023). Gut microbiota, comprising trillions of microorganisms, significantly influences systemic immunity and inflammation (Giambra et al., 2023; Li et al., 2023). Dysbiosis, an imbalance in the gut microbial composition, has been implicated in exacerbating psoriasis (Buhas et al., 2022; Cai et al., 2023; Du et al., 2023). Alterations in gut microbiota can lead to increased intestinal permeability, allowing bacterial products to enter the circulation and potentially trigger systemic inflammation, which can exacerbate skin lesions.

TCM, renowned for its holistic and integrative approach, has been utilized for centuries in the management of various dermatological disorders. Among the numerous TCM formulations, SLBZP stands out for its unique composition and therapeutic potential. SLBZP is a complex blend of several herbs, including ginseng and licorice, which are revered in TCM for their ability to strengthen the spleen and harmonize the gut, thereby promoting overall health and wellbeing. The efficacy of SLBZP in dermatological applications, especially in psoriasis, a chronic and inflammatory skin condition, has garnered attention in both traditional and modern medical spheres. This formulation is known for its potent anti-inflammatory properties, which are largely attributed to its diverse array of active constituents. These include polysaccharides, known for their immunomodulatory effects; saponins, which exhibit anti-inflammatory and antioxidant activities; and flavonoids, compounds recognized for their ability to scavenge free radicals and reduce oxidative stress.

Based on the research available on PubMed, studies related to SLBZP have primarily focused on its effects in different health conditions and its mechanisms of action, rather than directly linking it to dermatological applications or psoriasis. For instance, one study explored the mechanism of SLBZP in treating bronchial asthma and allergic colitis, highlighting the use of network pharmacology and molecular docking methods (Zeng et al., 2022). Another study indicated that SLBZP alleviates chronic inflammation, which can be associated with its potential effects on inflammatory skin disorders like psoriasis (Zhang et al., 2022b). Additionally, research has shown the hepatic protective effects of SLBZP, a factor that could indirectly influence skin health due to the liver’s role in systemic inflammation and metabolism (Pan et al., 2021). There’s also evidence of SLBZP affecting immunity in the context of diarrheal diseases, which points towards its immunomodulatory capabilities (Chen et al., 2022).

While these studies provide a basis for understanding the broad therapeutic applications of SLBZP, there seems to be a gap in the literature specifically focusing on its direct role in treating psoriasis through the modulation of lipid metabolism and the gut-skin axis. Therefore, in the context of your study, it would be novel to specifically emphasize SLBZP’s impact on lipid metabolism and its potential link to psoriasis treatment, supported by the understanding that SLBZP has general anti-inflammatory and immunomodulatory effects. Therefore, while the traditional use of SLBZP in TCM is well-documented, its application in modern medical practice, particularly for psoriasis treatment, represents an evolving area of scientific inquiry. Ongoing research into its pharmacological properties and mechanisms of action is crucial for integrating this ancient wisdom with contemporary medical understanding, potentially offering new avenues for the management of dermatological conditions.

This study posits that SLBZP may impart therapeutic benefits in the treatment of psoriasis predominantly via modulation of the gut microbiota. It theorizes that such modulation by SLBZP may lead to an attenuated inflammatory state, manifesting both systemically and within the cutaneous environment. Through this investigation, the study aims to delve into a relatively unexplored facet of psoriasis management, one that intricately interweaves the principles of traditional medicine with contemporary insights into the gut-skin axis. This integrative approach not only holds promise for unveiling novel strategies in the treatment of psoriasis but also contributes significantly to the burgeoning corpus of research underscoring the pivotal role of gastrointestinal health in systemic inflammatory disorders. By exploring this nexus, the study aims to illuminate new pathways in dermatological therapy, emphasizing the interconnectivity between internal organ systems and skin health.

## 2 Materials and methods

### 2.1 LC-MS conditions

The SLBZP was sourced from Guangzhou Baiyunshan Jingxiutang Pharmaceutical Co., Ltd., Guangzhou, China (Production Batch Number: 20211201). The chromatographic analysis utilized a Dionex Ultimate 3000 RSLC (HPG; Thermo Fisher Scientific, United States) with a Hypersil GOLD column (100*2.1 mm, 1.9 μm; Waters, United States). The mobile phase consisted of A: acetonitrile and B: 0.1% formic acid in water. The column temperature was maintained at 40°C, and the injection volume was 3 μL. A gradient elution at a flow rate of 0.2 mL/min was employed. The MS analysis was performed using a Thermo Scientific Q Exactive Focus (Thermo Fisher Scientific, United States) in full MS-ddms2 mode, scanning from 100 to 1,500 m/z. Additional MS scanning parameters are detailed in Supplementary Table S1.

### 2.2 Collection of active components and targets of SLBZP

Active components in SLBZP were collected using TCMSP, HERB, SymMap, and, ETCM databases, selecting those with oral bioavailability (OB) ≥ 30% and drug likeness (DL) ≥ 0.18. Targets from the databases were standardized and deduplicated using the Uniprot database to obtain the gene names of drug targets. The active components and their corresponding targets were finalized. The intersections of active components and targets among different drugs were visualized using Venn diagrams and Upset plots with R4.2.1 VennDiagram1.7.3 and UpSetR1.4.0 packages. The network pharmacology analysis was conducted online during the period from December 11 to 17 December 2023.

### 2.3 Selection of SLBZP drug targets related to psoriasis

Disease-related targets were retrieved from GeneCards, DisGeNET, DrugBank, and CTD databases using “psoriasis” as the keyword. These targets were then consolidated and standardized using the Uniprot database to normalize gene names and remove duplicates, resulting in potential disease targets. Venn diagrams were drawn to display the intersections of targets across different databases. The compound targets were intersected with psoriasis targets downloaded from databases in R Studio to identify common targets for drug treatment of psoriasis, which were used for subsequent analysis.

### 2.4 Drug-compound-target-disease network construction and analysis

The correspondence between active components and targets of SLBZP was imported into Cytoscape 3.9.1 software to construct a drug-compound-target-disease network diagram. In this network, nodes represent compounds and targets, while lines indicate the interactions between compounds and targets. The network’s topological properties were analyzed using the Network Analyzer plugin, calculating the degree value (degree) of each node.

### 2.5 Construction and analysis of protein-protein interaction network

Using the STRING database, a protein-protein interaction (PPI) network was constructed. Intersection targets related to psoriasis and acted upon by SLBZP were inputted into STRING, selecting “*Homo sapiens*” as the species and setting a confidence threshold of ≥0.9. This established the interaction relationships between proteins. The resulting data were saved as TSV and PNG files and imported into Cytoscape software. Isolated targets without intersections were removed, and the network’s topological properties were analyzed. Core targets were identified based on topology sorting (CC/BC/degree) and selected using the MCC algorithm of the cytohubba plugin.

### 2.6 Construction and analysis of the drug-compound-target-pathway-disease network

To uncover the potential mechanisms of the drug action, we selected core TOP pathways, key targets, and active components to construct a “Drug-Compound-Target-Pathway-Disease” network. This was done to better illustrate the interconnections between significant active components and targets of the drug and certain key pathways, demonstrating their synergistic effect.

### 2.7 GO functional enrichment and kyoto encyclopedia of genes and genomes pathway enrichment analysis

Targets related to psoriasis and SLBZP were subjected to Gene Ontology (GO) functional enrichment and Kyoto Encyclopedia of Genes and Genomes (KEGG) pathway enrichment analysis using the R clusterProfiler 4.6.2 package. GO enrichment analysis was conducted with a cutoff of *p* < 0.05, selecting the top 20 biological entries with *p*-values less than 0.05 for bar chart visualization. Similarly, KEGG enrichment analysis used a significance threshold of *p* < 0.05, with the top 30 pathways with *p* < 0.05 visualized in a bubble chart. Additionally, we employed the “pathview” package (Luo and Brouwer, 2013) in R to illustrate the lipid metabolism pathway.

### 2.8 Animal experiment

Mice were acquired from the Guangdong Medical Laboratory Animal Center, Guangzhou, China. A total of fifty specific pathogen-free (SPF) BALB/c mice were randomly allocated into five groups. Excluding the control group, each group was topically treated with 5% imiquimod (IMQ) cream (Med Shine; Sichuan, China), applying 62.5 mg/day for 7 consecutive days. Concurrently, the SLBZP group received an oral administration of SLBZP suspension at a dose of 0.86 g/kg and 1.72 g/kg. Methotrexat (MTX) (1 mg/kg) and was administered as positive control. Following the intervention period, mice were humanely euthanized in accordance with established animal protocols. All experimental procedures were meticulously executed in line with the stringent guidelines set forth by the institution’s animal experiment center. To evaluate the severity of the induced psoriatic-like lesions in mice, a modified psoriasis area and severity index (PASI) scale was utilized (Zecevic-Pasic et al., 2023). This scale incorporated parameters such as erythema, scaling, and epidermal thickening. The PASI scores were stratified as follows: 0 (absent), 1 (mild), 2 (moderate), 3 (severe), and 4 (extremely severe).

### 2.9 H&E staining and IHC staining

Mice were euthanized after treatment, and approximately 1 cm^2^ of dorsal skin was excised and fixed in a buffered 4% paraformaldehyde solution. The tissue was then embedded in paraffin, sectioned at a thickness of 5 μm, and subjected to hematoxylin-eosin staining for histological examination. For immunohistochemical (IHC) analysis, sections were incubated with primary antibodies: rabbit anti-mouse Ki67 (1:500; Abcam, United States) and rabbit anti-mouse interleukin (IL)-17 (1:200; Abcam, United States). This was followed by application of a secondary antibody reagent kit (Maxim; Fuzhou, China) and visualization using DAB substrate (BD Pharmingen, United States). After counterstaining with hematoxylin, the sections were dehydrated, cleared, and mounted on glass slides for observation and measurement under an upright microscope (Olympus Corporation, Japan).

### 2.10 Fecal DNA extraction and 16S rRNA sequencing

The stools of mice were sent to Major Bioengineering (Shanghai) Co., Ltd. for 16S rRNA sequencing. Total microbial genomic DNA was extracted from stool samples using the Mag Atrract Power Soil Pro DNA Kit (Qiagen, Hilden, Germany) according to manufacturer’s instructions. The quality and concentration of DNA were determined by 1.0% agarose gel electrophoresis and a NanoDrop^®^ ND-2000 spectrophotometer (Thermo Scientific Inc., United States) and kept at −80 °C prior to further use. The hypervariable region V3-V4 of the bacterial 16S rRNA gene were amplified with primer pairs 338F (5′-ACT​CCT​ACG​GGA​GGC​AGC​AG-3′) and 806R (5′-GGACTACHVGGGTWTCTAAT-3′) by an ABI GeneAmp^®^ 9700 PCR thermocycler (ABI, CA, United States). The PCR reaction mixture including 4 μL 5 × Fast Pfu buffer, 2 μL 2.5 mM dNTPs, 0.8 μL each primer (5 μM), 0.4 μL Fast Pfu polymerase, 10 ng of template DNA, and ddH_2_O to a final volume of 20 µL. PCR amplification cycling conditions were as follows: initial denaturation at 95 °C for 3 min, followed by 29 cycles of denaturing at 95 °C for 30 s, annealing at 53 °C for 30 s and extension at 72 °C for 45 s, and single extension at 72 °C for 10 min, and end at 4 °C. All samples were amplified in triplicate. The PCR product was extracted from 2% agarose gel and purified. Then quantified using Quantus™ Fluorometer (Promega, United States).

### 2.11 Alpha diversity and beta diversity analysis

In the present study, alpha diversity was rigorously evaluated utilizing the Sob, Chao1, Shannon, and Simpson indices. The Chao1 estimator was employed to ascertain the richness of the bacterial community. The Shannon diversity index encapsulated both community richness and evenness, while the Simpson index provided insight into overall community diversity. For assessing beta diversity, both principal coordinate analysis (PCoA) and nonmetric multidimensional scaling (NMDS) analyses were meticulously applied. These analytical methodologies facilitated the reduction of the dimensionality inherent in multi-dimensional microbial datasets, illuminating the primary trajectories of data variance as depicted along continuous sequencing axes.

### 2.12 Species composition and difference analysis

Following taxonomic analysis, we meticulously examined the species composition of each sample group at genus level. Utilizing community bar charts, we were able to provide a visual representation of the predominant species within each sample group, delineating the relative abundance (proportion) of each dominant species. Leveraging the derived community abundance data, advanced statistical methodologies were employed to assess the hypothesis concerning species distribution among microbial communities within each group. This approach facilitated the evaluation of the significance levels pertaining to species abundance disparities at the phyla and genus taxonomic tiers, thereby pinpointing species exhibiting marked differences between groups.

### 2.13 Statistical analyses

Enumeration data were expressed as frequencies and percentages. The Shapiro-Wilk normality test and Levene’s homogeneity of variance test were performed first. The Kruskal-Wallis rank-sum test was used for multiple group comparison when the data did not conform to a normal distribution. The Bonferroni test was used for the homogeneity of variance, and Dunnett’s T3 test was used for the heterogeneity of variance. In the case of normal distribution and homogeneity of variance, a one-way analysis of variance (ANOVA) Bonferroni test was used for the comparison of sample means between multiple groups. *p* < 0.05 was considered statistically significant. All data were analysed using SPSS28.0 (SPSS Inc., Chicago, Illinois, United States).

## 3 Results

### 3.1 The qualitative identification of main chromatographic peaks of SLBZP was established

**FIGURE 1:**
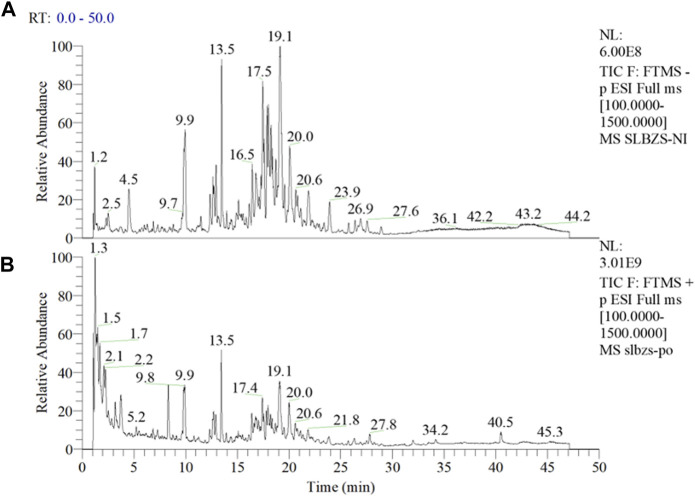
Total Ion Chromatogram of SLBZP Capsule Samples **(A)** represents the negative ion mode, illustrating a higher number of detectable medicinal components, with peaks labeled by their retention times in minutes. **(B)** represents the positive ion mode, depicting a fewer number of components. Analysis by Compound Discoverer software, supplemented with manual verification, identified 42 principal components and potential quality markers, primarily derived from licorice and ginseng, such as flavonoids, saponins, and ginsenosides, along with other significant markers from various medicinal materials. For detailed information on the identified components and quality markers, refer to Supplementary Table S2.

As evident from Figure 1, a larger number of medicinal components were detected under negative mode (Figure 1A), while only a few were detected under positive mode (Figure 1B). Utilizing the Compound Discoverer software for analysis, complemented by manual inspection, we organized a total of 42 principal components and potential quality markers of the compound formulation (Supplementary Table S2), of which 41 have been identified. The main constituents originated from formula components such as licorice and ginseng, including flavonoids and saponins from licorice, and ginsenosides from ginseng. Quality marker components suitable for quality control from other medicinal materials included triterpenic acids from Poria (Dehydrotumulosic acid, Tumulosic acid, Polyporenic acid C, Pachymic acid), sesquiterpenes from Atractylodes (atractylenolide III, atractylenolide II, atractylenolide I), saponins from Platycodon (Platycodin J/Platyconic acid A, Platycodin A), and alkaloids from lotus seeds (Neferine), with 12 components currently confirmed through standard reference comparison (See Supplementary Table S2 for detailed information). ZP Capsule Samples (A: Negative Mode; B: Positive Mode).

### 3.2 The collection and prediction of targets related to active pharmaceutical ingredients and psoriasis

**FIGURE 2:**
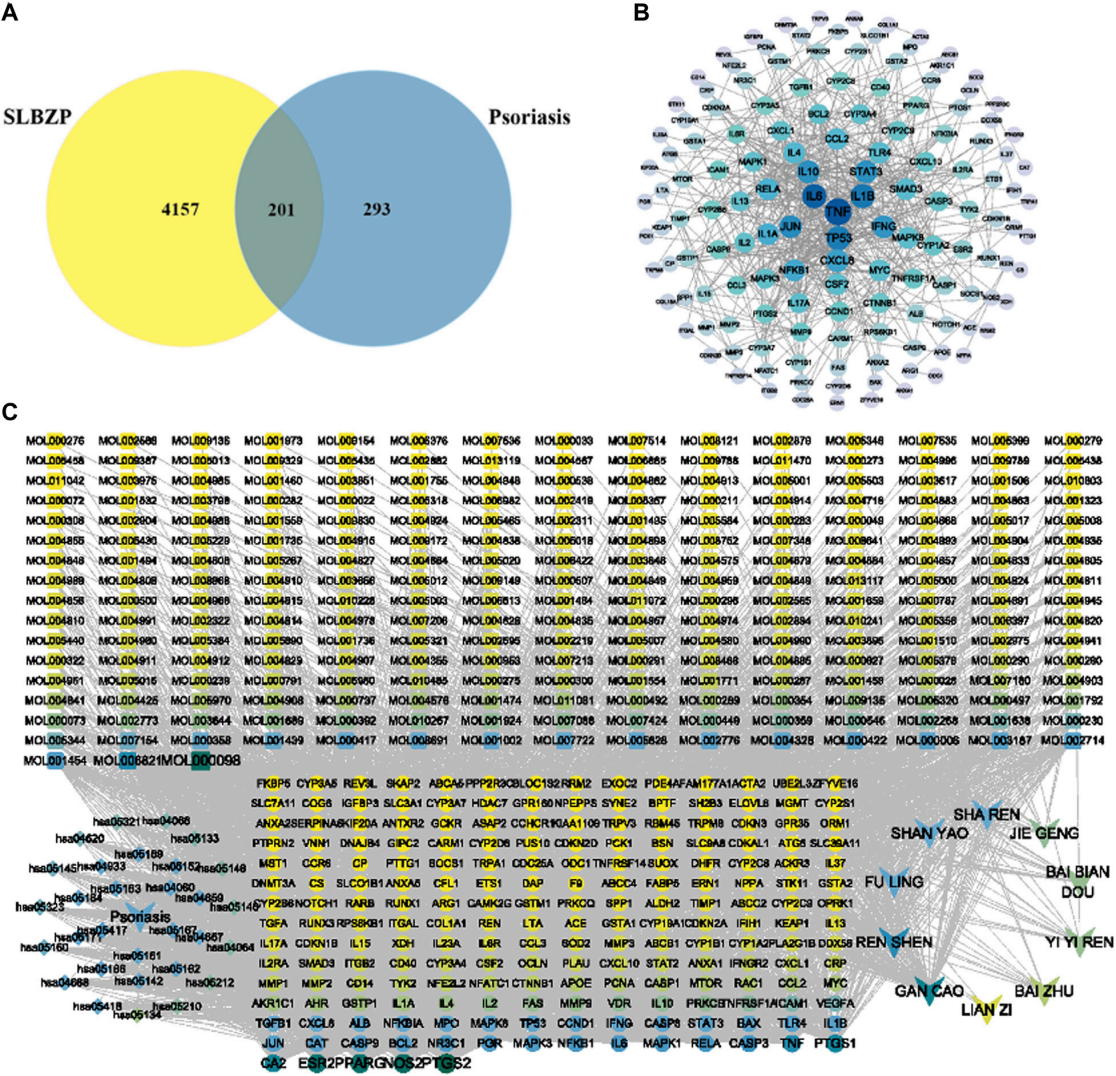
Integrative Network Analysis of SLBZP: Target Identification and Pathway Interaction in Psoriasis Treatment **(A)** displays the intersection of SLBZP components and psoriasis targets, identifying 201 common targets. **(B)** visualizes the protein-protein interaction network of these targets. **(C)** presents the ‘Compound-Target-Pathway-Disease’ network, showcasing the interaction between SLBZP’s components, psoriasis targets, and related pathways, underlining the formulation’s multi-target therapeutic potential.

Active components of SLBZP were identified using pharmaceutical databases, selecting those with OB ≥ 30% and DL ≥ 0.18. After removing duplicate common components, a total of 259 active components were identified. A single herb may contain multiple active components, and the same component can be derived from different herbs, reflecting the synergistic mechanism of multi-component, multi-target action in traditional Chinese medicine. Using the SwissTargetPrediction database, potential targets of the active components were predicted. After eliminating duplicate targets, a total of 4358 drug targets were identified. Psoriasis-related targets were collected using thresholds set in various databases: GeneCards Score ≥10, DisGeNET Score ≥0.1, DrugBank, and CTD Score ≥30. After selection and standardization, 494 disease targets were identified. Potential disease targets were intersected with the 4358 targets of SLBZP’s active components. This intersection resulted in 201 common targets, identified as potential action targets of the drug for psoriasis, as illustrated in Figure 2A. The 201 intersecting targets related to the treatment of liver fibrosis by the drug were imported into the STRING database to obtain PPI relationships. These were then analyzed in Cytoscape software. The Network Analyzer plugin was used to analyze network topological properties and calculate degree values, with the intensity of color and node size representing the magnitude of the degree values. The top 30 core nodes were selected based on degree value (CC/BC/degree) rankings, and a target degree value ranking chart (Figure 2B) was created. Key targets identified included tumor necrosis factor-alpha (TNF-α), IL-6, IL-1β, TP53, JUN, etc., serving as critical targets for SLBZP in the treatment of psoriasis. We selected the core pathways along with key targets and active components to construct a “Compound-Target-Pathway-Disease” network. As shown in Figure 2C, this network comprises 485 nodes and 3025 edges (including 243 components, 201 targets, 30 signal pathways, the drug, and the disease). The nodes are not uniformly distributed; rather, there are denser clusters that may signify key subnetworks or pathways with a higher degree of interaction. Notably, some nodes and pathways are labeled more prominently, which may highlight their relative importance or the strength of evidence supporting their role in psoriasis. The map serves as a comprehensive framework, illustrating the intricate network of molecular interactions associated with psoriasis, and may be utilized to identify novel therapeutic targets or to understand the multifactorial nature of the disease. This kind of network is instrumental for researchers to synthesize large volumes of data and to conceptualize the multifaceted interactions that occur within biological systems in health and disease states.

### 3.3 GO functional enrichment and KEGG pathway analysis

**FIGURE 3:**
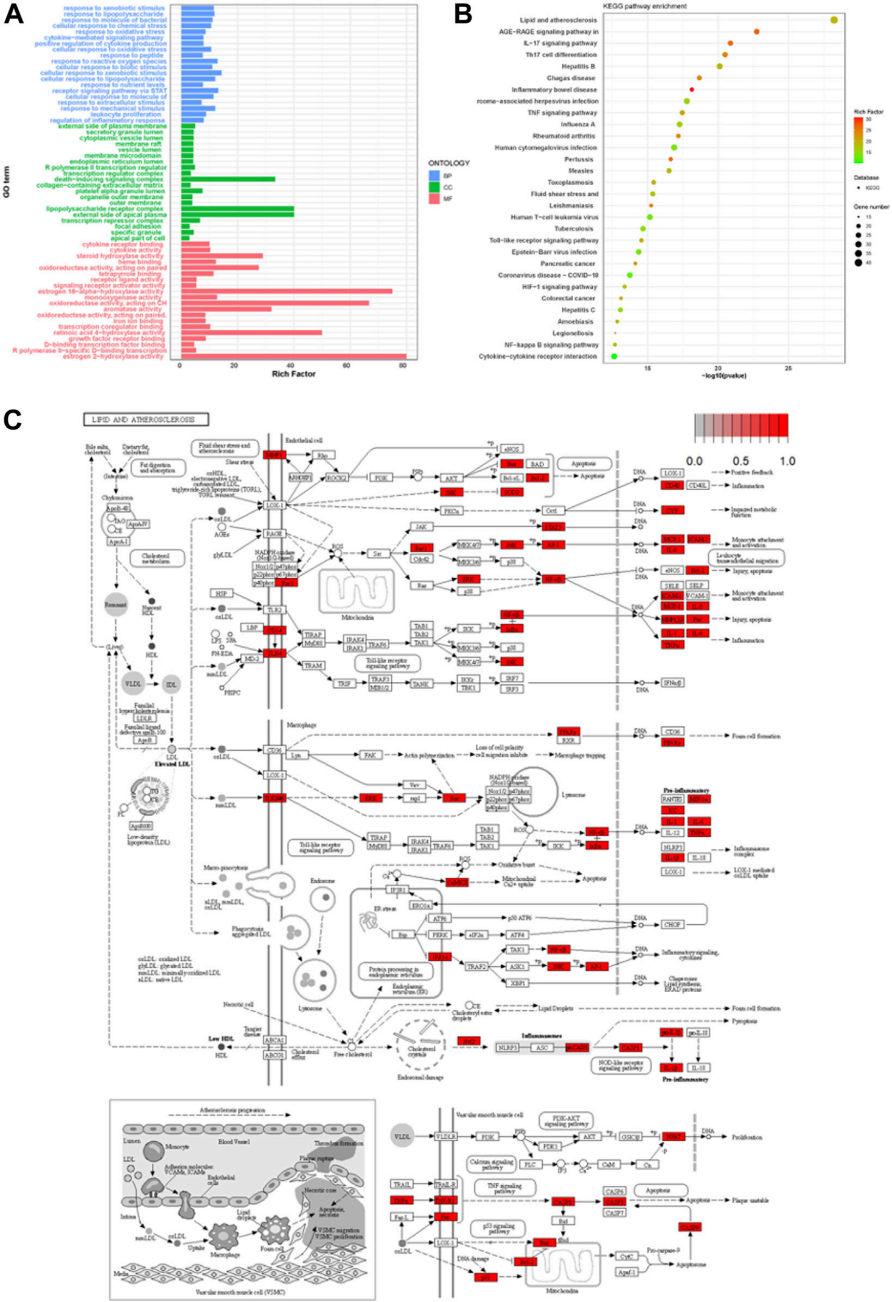
Comprehensive Analysis of Psoriasis Treatment Targets: GO Enrichment, KEGG Pathway Integration, and Lipid Metabolism Interactions **(A)** displays the GO enrichment analysis of the top 20 biological processes, molecular functions, and cellular components associated with 201 psoriasis treatment targets. **(B)** visualizes the top 30 enriched KEGG pathways, highlighting key pathways involved in lipid metabolism and inflammation. **(C)** depicts the KEGG pathway map, illustrating the complex interactions within lipid metabolism that contribute to inflammatory processes in psoriasis.

Using R clusterProfiler, 201 intersecting targets related to the treatment of psoriasis were subjected to GO enrichment analysis. The top 20 entries with *p* < 0.05 in biological process (BP), molecular function (MF), and cellular component (CC) were selected for bar chart visualization, as shown in Figure 3A. The *y*-axis represents various GO entries, and the *x*-axis indicates the number of targets enriched in each entry. BP primarily involved responses to external stimuli, responses to lipopolysaccharides, responses to bacterial molecules, etc.; MF included cytokine receptor binding, receptor ligand activity, signal receptor activator activity, etc.; CC mainly encompassed the external side of the plasma membrane, transcriptional regulation complex, secretory granule lumen, etc. KEGG pathway enrichment analysis of the targets yielded 168 signal pathways in total. For this study, the top 30 pathways with *p* < 0.05 from the download results were selected for bubble chart visualization, as depicted in Figure 3B. The enrichment results primarily involved pathways such as lipid and atherosclerosis, AGE-RAGE signaling pathway, IL-17 signaling pathway, Th17 cell differentiation, etc., with pathways synergistically contributing to the treatment of psoriasis. KEGG pathway map (Figure 3C) delineated the molecular interactions and reactions involved in lipid metabolism, suggesting a complex interaction between lipid metabolism and inflammatory processes.

### 3.4 Efficacy of different treatments in psoriasis mice models

**FIGURE 4:**
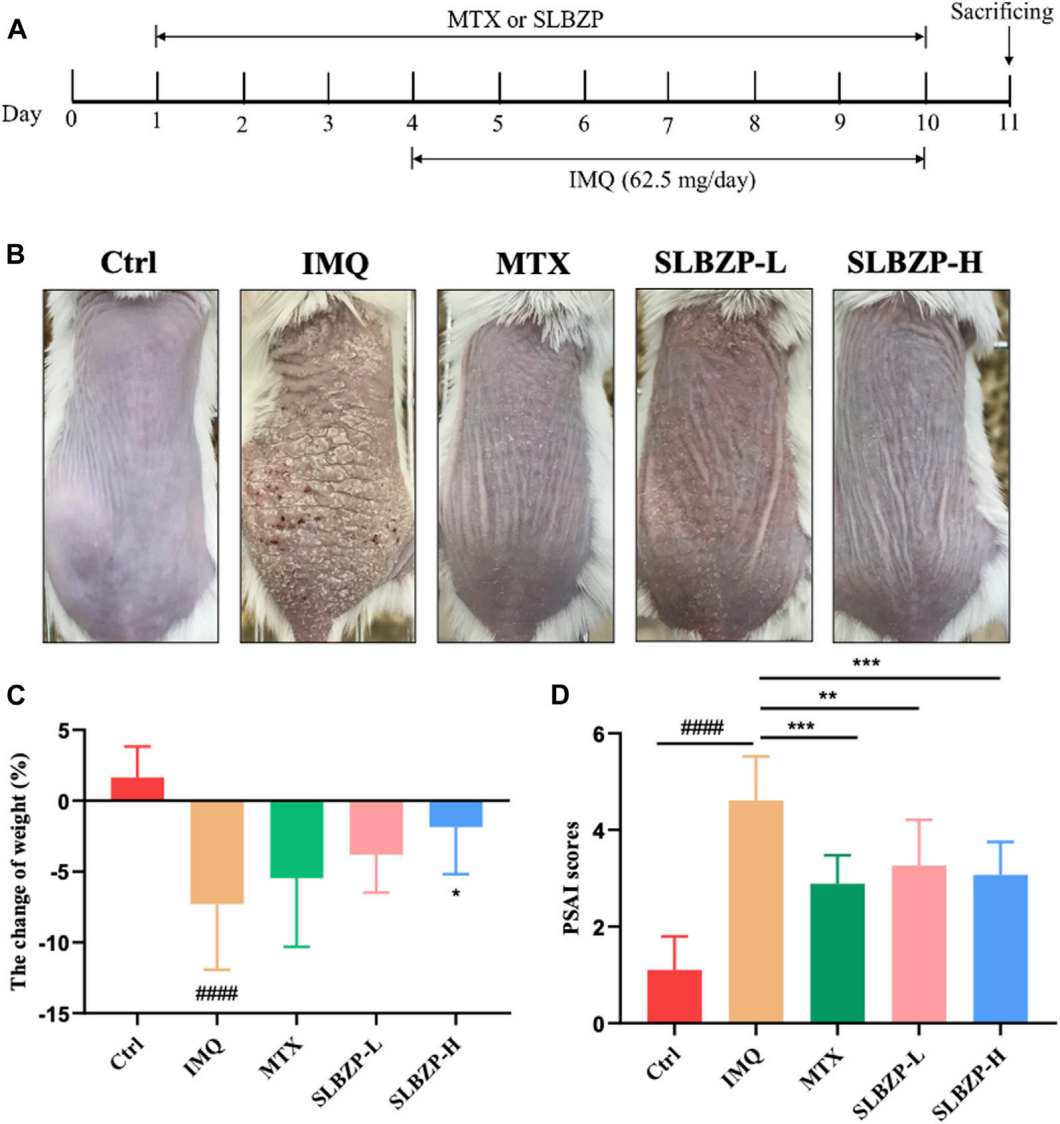
Therapeutic Efficacy of MTX and SLBZP in Psoriasis Treatment: A Multi-Parameter Evaluation **(A)** details the 11-day treatment regimen with MTX or SLBZP and daily IMQ application to induce psoriasis-like skin lesions. **(B)** visually compares the skin response to treatments, showing reduced irritation in the MTX and SLBZP groups *versus* the IMQ group. **(C)** graphs weight changes, indicating protection against IMQ-induced weight loss by MTX and SLBZP. **(D)** presents the PASI scores, with lower scores in MTX and SLBZP groups, suggesting alleviated symptom severity.

In the comprehensive assessment of different treatments for psoriasis mice models, the study presents a clear timeline of treatment application with MTX and SLBZP across 11 days, accompanied by daily IMQ application to induce symptoms (Figure 4A). The visual comparison showcases the skin’s response to each treatment, with less irritation in MTX and SLBZP groups compared to the IMQ group (Figure 4B). The weight change analysis indicates that while IMQ administration led to weight loss, MTX and SLBZP treatments appeared to prevent this loss (Figure 4C). Finally, the PASI scores, which reflect the severity of psoriasis symptoms, were notably lower in the MTX and SLBZP-treated groups, suggesting a reduction in symptom severity (Figure 4D).

### 3.5 H &E staining and immunohistochemical staining analysis

**FIGURE 5:**
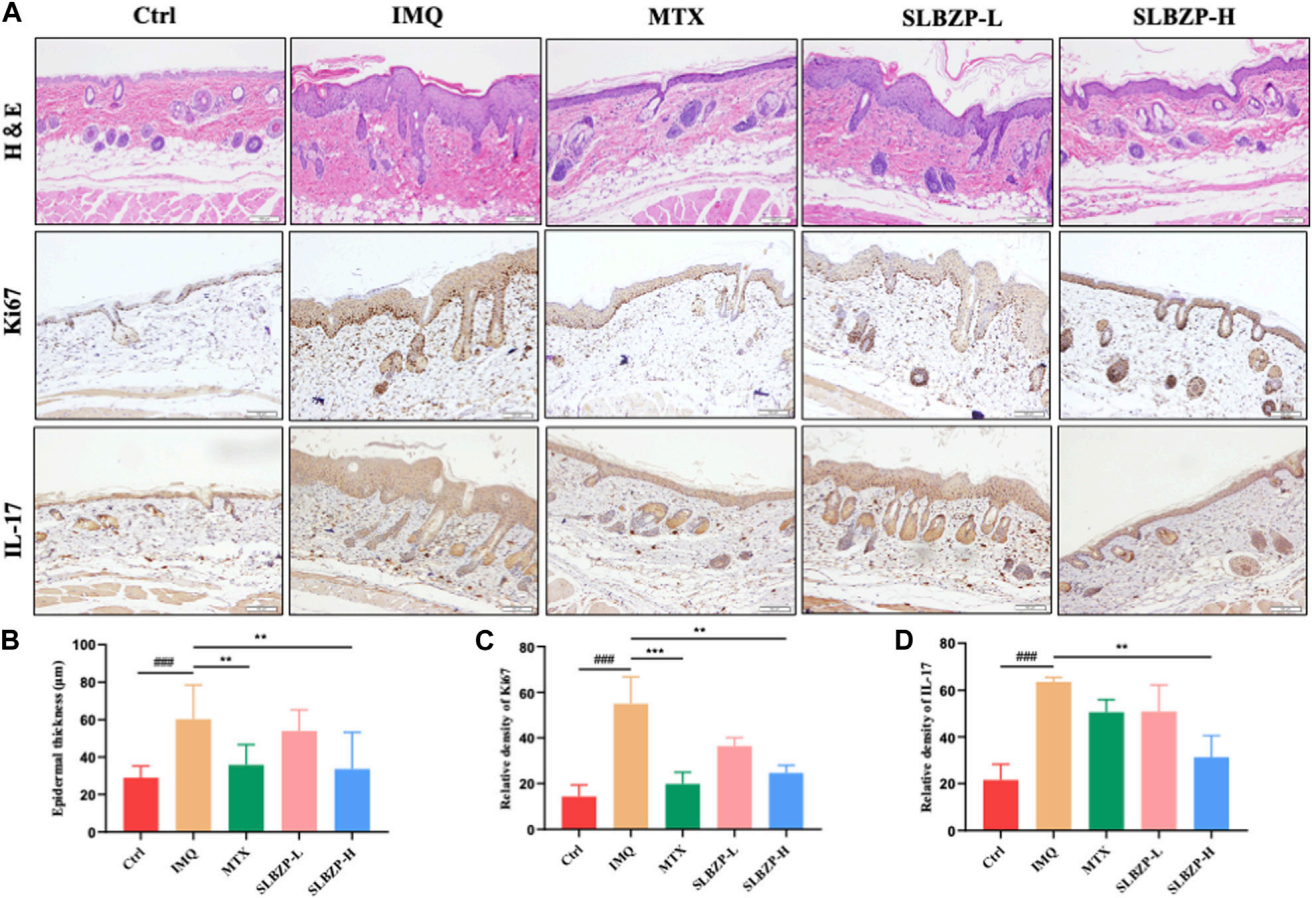
Histopathological and Immunohistochemical Assessment of Psoriasis Treatment in Mice with SLBZP and Methotrexate **(A)** presents H&E, Ki67, and IL-17 staining, demonstrating tissue structure, cell proliferation, and inflammation levels across treatment groups. **(B–D)** quantify epidermal thickness, cell proliferation, and IL-17 expression, with SLBZP-H showing the most pronounced reduction in psoriatic symptoms compared to IMQ and MTX.).

The combined histological and immunohistochemical evaluation of psoriasis treatments in mice is presented in Figure 5. Figure 5A shows tissue staining, with H&E indicating tissue structure and cellularity, Ki67 marking cell proliferation, and IL-17 denoting inflammation. Treatment groups, particularly SLBZP-H, exhibit reduced epidermal thickening, proliferation, and inflammation compared to the IMQ group. The quantified data in Figures 5B–D reflect these observations, with SLBZP-H demonstrating the most significant improvements. These findings collectively suggest that SLBZP, especially at higher doses, effectively alleviates psoriatic features in this model, potentially offering a comparable alternative to methotrexate.

### 3.6 Alpha and beta diversity analysis

**FIGURE 6:**
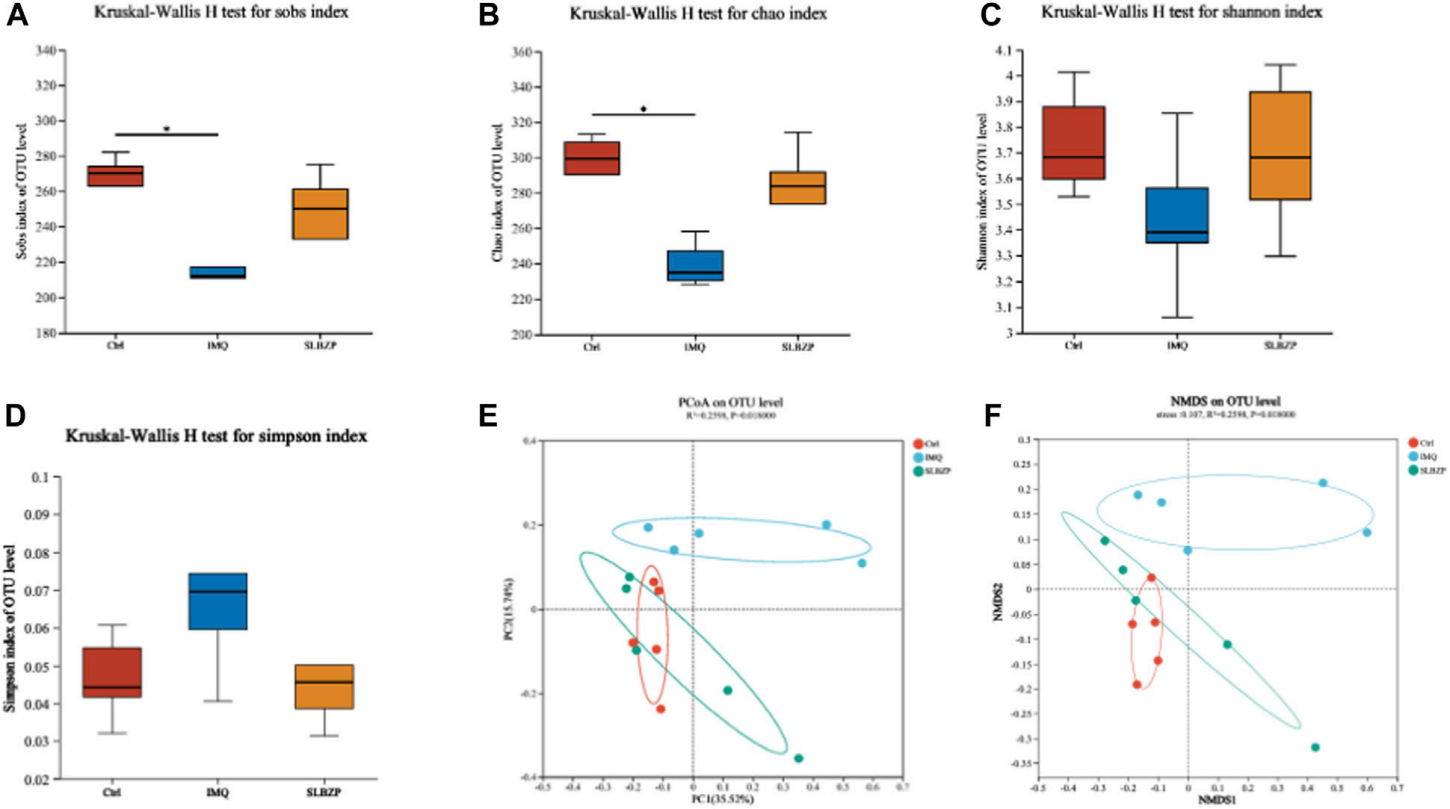
Alpha and Beta Diversity Analysis of Gut Microbiota in Psoriasis: Effects of SLBZP Treatment **(A–C)** depict alpha diversity metrics (Sobs index, Chao1, Shannon index), indicating enhanced microbial diversity with SLBZP treatment compared to the IMQ group. **(D)** shows the Simpson index, further supporting increased diversity post-treatment. **(E, F)** visualize beta diversity, demonstrating the shift in microbiota composition towards the control profile with SLBZP treatment, suggesting its restorative effects on microbiota disrupted by IMQ.

In Figure 6, the comprehensive analysis of alpha and beta diversity of the gut microbiota across the control, IMQ model, and SLBZP treatment groups is presented. Alpha diversity indices, including species observed (Sobs), Chao1, and Shannon diversity, are depicted in Figures 6A–C, showing that SLBZP treatment appears to enhance microbial richness and evenness compared to the IMQ group. The Simpson index (Figure 6D) corroborates these findings, indicating a trend towards increased diversity with SLBZP treatment. Beta diversity, illustrating the differences in microbial communities between the groups, is shown in Figures 6E,F, where the SLBZP group’s microbiota composition demonstrates a shift towards the control group’s profile, away from the IMQ model’s altered state. This suggests a restorative effect of SLBZP on gut microbiota diversity after IMQ-induced disruption.

### 3.7 Impact of SLBZP on the composition and potential functional aspects of the gut microbiota within a psoriasis model

**FIGURE 7:**
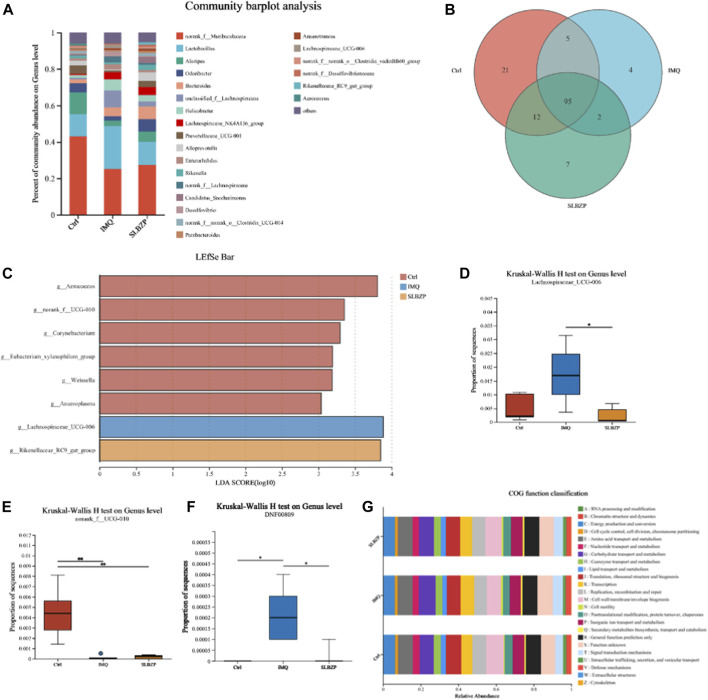
Comparative Microbial Community Analysis in Psoriasis: Influence of SLBZP Treatment on Gut Microbiota Diversity and Function **(A)** displays a community barplot comparing the gut microbiota composition across control, IMQ, and SLBZP-treated groups. **(B)**’s Venn diagram shows the unique and shared microbial species among the groups. The LEfSe bar chart **(C)** identifies bacterial genera with significant differences between the groups. Box plots **(D–F)** detail the abundance of specific bacterial genera, indicating changes in microbiota related to each treatment. **(G)** provides a COG functional classification of the gut microbiota, elucidating the potential metabolic impact of the treatments.

The results from the gut microbiota analysis in control, IMQ, and SLBZP treatment groups in mice are detailed in Figure 7. The community barplot (Figure 7A) delineates distinct microbiota profiles among the three groups, indicating that both IMQ and SLBZP treatments exert an influence on the gut microbial community structure. The Venn diagram (Figure 7B) visualizes the distribution of microbial species across the three distinct mouse groups. It shows that the control group harbors 21 unique species, the IMQ group possesses 4 unique species, and the SLBZP group contains 7 unique species. A core microbiome comprising 95 species shared across all groups is identified, underlining a common microbial baseline. Additionally, the diagram reveals specific overlaps in microbial species between groups: 12 species between the control and SLBZP groups, 5 between control and IMQ groups, and 2 between IMQ and SLBZP groups.

The Linear Discriminant Analysis Effect Size (LEfSe) bar chart (Figure 7C) highlights the bacterial genera that distinguish the differences between the groups. Each bar represents the Linear Discriminant Analysis (LDA) score for a specific bacterial genus, indicating its relative impact in differentiating the microbial communities of each group. The chart depicts a clear distinction in the microbial profile among the three conditions. The taxa such as *g__Aerococcus* and *g__norank_f__UCG-010* demonstrate a higher abundance in the SLBZP condition compared to the control, as indicated by the extended red bars. Conversely, *g__* *Lachnospiraceae* *_UCG-006* and *g__*Rikenellaceae*_RC9_gut_group* are more abundant in the IMQ condition, which is denoted by the prominent blue bars.

The box plots (Figures 7D–F) present the results of the Kruskal-Wallis H test at the genus level for *Lachnospiraceae* *UCG-006*, *norank_f__UCG-010*, and *DNF00809* across the three groups. For *Lachnospiraceae* *UCG-006*, the IMQ group exhibits a significantly higher median proportion compared to the control and SLBZP groups, with the SLBZP group showing a reduction towards control levels. In the case of *norank_f__UCG-010*, the control group demonstrates a higher median proportion, whereas the IMQ and SLBZP groups show a notably lower abundance. For *DNF00809*, the IMQ group presents a markedly higher median proportion in contrast to both the control and SLBZP groups.

Finally, the bar chart (Figure 7G) displays the Cluster of Orthologous Groups (COG) functional classification of the gut microbiota across the three groups. Each bar in the chart represents the relative abundance of microbial genes associated with diverse biological functions, such as energy production, amino acid metabolism, and lipid transport, highlighting how treatments potentially influence the metabolic capabilities of the gut microbiota.

## 4 Discussion

In this study, the effects of SLBZP, a traditional Chinese medicine, on psoriasis and gut microbiota were evaluated in mice models. Through LC-MS, 42 primary components, mainly flavonoids and saponins from licorice and ginseng, were identified. Network pharmacology revealed 259 active components targeting pathways in psoriasis, focusing on TNF, IL-6, and IL-1β. Additionally, KEGG pathway analysis highlighted the importance of lipid metabolism in psoriasis treatment, showing complex interactions between lipid metabolism and inflammation. The experimental results in a psoriasis rat model indicated SLBZP’s significant therapeutic effects, marked by changes in skin pathology and inflammatory biomarkers, as well as notable alterations in gut microbiota composition, particularly in microbial abundance and diversity. This suggests SLBZP’s potential role in modulating the gut-skin axis, with shifts in anti-inflammatory microbial genera like *Lachnospiraceae* *UCG-006* and *norank_f__UCG-010*.

The integration of empirical and computational methods is crucial in enriching our understanding of the pharmacological profile of SLBZP. This multidimensional approach leverages the strengths of both methodologies to provide a more comprehensive and nuanced perspective of SLBZP’s active components and their potential therapeutic effects. While the empirical data provide a solid foundation and validation for the study, the computational analysis offers a broader view of the potential pharmacological landscape. This combination allows us to hypothesize more confidently about the therapeutic effects of SLBZP, including potential mechanisms of action and interactions with biological systems.

Recent advancements in the field of lipid metabolism and inflammation have elucidated their critical interplay and its implications for a variety of diseases. Lipid metabolism is essential in modulating both acute and chronic inflammation, with dietary and endogenous lipids displaying properties that can either promote or mitigate inflammatory responses. This modulation significantly impacts atherogenic and immunomodulatory pathways (Andersen, 2022). Inflammation and infections are known to induce alterations in lipid metabolism, which may initially act to suppress inflammation or combat infection. However, when these changes become chronic, they contribute to an increased risk of atherosclerosis, often characterized by a decrease in serum HDL and an increase in triglycerides.

Furthermore, there is a recognized coupling between lipid metabolism and inflammation, suggesting a substantial crosstalk between these two processes. Despite the accumulating evidence of this interaction, the specific molecular mechanisms operating under both physiological and pathophysiological conditions remain elusive and poorly understood (Zhang et al., 2022a). This lack of clarity extends to the field of autoimmune rheumatic diseases (AIRDs), where lipid metabolism influences both the inflammation process and the mechanisms of action of AIRD therapeutics. Opportunities are being explored for co-therapies targeting lipid metabolism that could mitigate immunometabolic complications and potentially reduce the increased cardiovascular disease risk in patients with AIRDs (Robinson et al., 2022).

Additionally, dysregulation in lipid metabolism directly influences inflammatory processes and is a key factor in the etiology of several diseases, including cardiovascular disease and diabetes. These conditions are often triggered or exacerbated by chronic inflammation (Anand, 2020). The intricate relationship between lipid metabolism and inflammatory processes underscores its significance in disease pathogenesis and the potential for targeted therapies and preventive strategies in conditions associated with dysregulated lipid metabolism and inflammation. As research continues to unravel the complexities of this relationship, it holds the promise of informing new therapeutic approaches and enhancing our understanding of the underlying mechanisms of a wide range of inflammatory and metabolic disorders.

Recent research in the field of lipid metabolism and psoriasis has provided critical insights into the complex relationship between these two elements. Studies have shown that systemic inflammation in psoriasis leads to an increased accumulation of pro-inflammatory oxidized lipids in the skin, derived predominantly from omega-6 fatty acids. These are counterbalanced by anti-inflammatory lipid mediators from omega-3 polyunsaturated fatty acids (Sorokin et al., 2020). This dynamic indicates a significant interplay between different types of lipids in the inflammatory pathways of psoriasis.

Additionally, there is a strong association between alterations in cholesterol and lipid metabolism with psoriasis, established through decades of preclinical and clinical studies. Key cytokines involved in psoriasis, such as TNF-αand IL-17, are known to affect cholesterol and lipid metabolism, suggesting a direct link between lipid dysregulation and the pathogenesis of psoriasis (Luo et al., 2023). Furthermore, psoriasis shares several commonalities with atherosclerosis, including the involvement of specific cytokines in their immunological mechanisms (such as IL-17), common angiogenic factors, and oxidative pathways. This connection underscores the influence of lipid metabolism imbalance in both conditions (Shih et al., 2020).

Research has also delved into the metabolic influences on T cells in psoriasis, examining how psoriasis affects the regulation of metabolites in glucose metabolism, lipid metabolism, amino acid metabolism, and other pathways within T cells. This highlights the systemic nature of psoriasis, affecting various metabolic processes beyond the skin (Su et al., 2023). Abnormal lipid distribution was also observed in patients with psoriasis increases their risk for atherosclerosis. This has led to investigations into the role of lipid-lowering drugs in the treatment of psoriasis, indicating their potential therapeutic effects, which is consistent with our findings. However, comprehensive evidence-based medical evaluations in this area are still lacking (Wang et al., 2022).

In summary, these studies collectively highlight the critical role of lipid metabolism in psoriasis, offering new perspectives for understanding the disease’s pathogenesis and potential therapeutic approaches. This emerging evidence suggests that targeting lipid metabolism could be a viable strategy in managing psoriasis and its associated risks.

On the other hand, recent research has also significantly advanced our understanding of the interplay between gut microbiota, lipid metabolism, and inflammation. The gut microbiome, comprising a diverse community of bacteria, plays a pivotal role in various functions that influence host health. These functions include nutrient metabolism, immune system regulation, and natural defense against infection (Al Bander et al., 2020). The metabolites produced by commensal gut microbes significantly impact host health through their interaction with the immune system and their influence on a multitude of metabolic pathways (Brown et al., 2023).

A significant area of focus is the gut microbiota’s role in lipid and lipoprotein metabolism. The intricate relationship between the gut microbiota and host metabolism is evident, particularly in how microbial populations and their metabolic activities relate to lipid metabolism. This relationship is crucial in understanding the pathophysiology of various metabolic and inflammatory conditions (Yue et al., 2019). Moreover, studies have highlighted the importance of the gut microbiome in lipid metabolism and its subsequent impact on host physiology. The complex interactions between the gut microbiota and the host’s immune and metabolic systems are central to the body’s overall health and disease state (Brown et al., 2023). Taken together, the emerging research underlines the gut microbiota’s integral role in lipid metabolism and inflammation. Understanding these complex interactions offers potential therapeutic targets for treating a range of metabolic and inflammatory disorders, emphasizing the importance of maintaining a healthy gut microbiome.

Recent studies on SLBZP have focused on its effects on lipid metabolism, inflammation, and gut microbiota, revealing its therapeutic potential in various conditions. One study evaluated the effects of SLBZP on gut microbiota and antioxidation in a rat model of ulcerative colitis (UC). This study aimed to understand how SLBZP supplementation could influence gut health and oxidative stress in inflammatory bowel conditions (Gu et al., 2021). SLBZP has been demonstrated to exert protective effects against non-alcoholic fatty liver disease (NAFLD). However, the specific mechanisms behind these effects are not fully understood. One study undertook lipidomic analysis to uncover these mechanisms, indicating SLBZP’s potential role in managing lipid-related disorders (Deng et al., 2019; Pan et al., 2019). Another study explored the role of SLBZP in the prevention and treatment of type 2 diabetes, focusing on flora disorder and chronic inflammation. This study aimed to understand how SLBZP’s modulation of gut microbiota could impact chronic inflammation and metabolic disorders like diabetes (Zhang L. J. et al., 2022). SLBZP is known to positively affect the metabolism of gut microbiota. While the exact mechanisms of how microbiota metabolites mediate anti-inflammation signaling are not fully elucidated, previous research has shown that SLBZP supplementation can alleviate conditions like antibiotic-associated diarrhea (Lv et al., 2022). These studies collectively indicate that Shenling Baizhu San has a multi-faceted impact on health, particularly in relation to lipid metabolism, gut microbiota balance, and inflammatory processes. Its role in managing conditions like NAFLD and type 2 diabetes highlights its potential for broader application in metabolic and inflammatory diseases. However, further research is necessary to fully elucidate the underlying mechanisms of its therapeutic effects.

The concept of the “gut-skin axis” underscores the bidirectional relationship between the gut microbiome and skin health. Recent studies in this field, particularly focusing on conditions like psoriasis, reveal how gut microbiota modulation can influence the development of skin disorders. This relationship is mediated through various mechanisms, including inflammatory mediators and the immune system. Research has demonstrated that the gut microbiome plays a critical role in the modulation of diseases beyond the gastrointestinal tract, including various skin disorders such as psoriasis, atopic dermatitis, rosacea, acne vulgaris, photoaging, and cutaneous wounds (Sinha et al., 2021). This connection is regulated through several pathways, including the immune system and the release of various inflammatory mediators (Thye et al., 2022).

The emerging research on the gut-skin-brain axis further highlights the intricate relationship between dietary habits, intestinal microbiota, and their impact on the development of not only intestinal and skin diseases but also neurological, psychiatric, and psychological disorders (Ferraretto et al., 2023).

Specifically, in the context of psoriasis, there is an increasing focus on understanding the disease from the perspective of the gut. This approach is opening new avenues for research and the development of novel therapeutics targeted at this complex interplay (Su et al., 2021). In the context of SLBZP, its effects on lipid metabolism and inflammation, as well as its influence on gut microbiota, lend support to the gut-skin axis hypothesis. The modulation of gut microbiota by SLBZP, particularly its impact on lipid metabolism and inflammatory processes, can have a significant effect on skin health, including the management of conditions like psoriasis. This aligns with the current understanding of the gut-skin axis, where intestinal health directly impacts skin condition and *vice versa*, mediated through metabolic and inflammatory pathways.

This study primarily focused on predictive analyses using network pharmacology and metagenomics, yet the examination of inflammatory markers was comparatively limited. The current research lacks in-depth experimental exploration, both *in vitro* and *in vivo*, to validate the mechanisms involving lipid metabolism and inflammation pathways. Addressing these gaps will be the primary objective of our subsequent research efforts.

In conclusion, this research has successfully integrated component analysis, network pharmacology, experimental validation, and gut microbiota studies to establish the lipid metabolism pathway as the key target for SLBZP in treating psoriasis. It delves into the potential interplay between SLBZP’s regulatory effect on gut microbiota, its influence on lipid metabolism, and the consequent mitigation of psoriasis symptoms. We propose that SLBZP’s ability to rebalance the gut microbiota might lead to a reduction in inflammation mediators linked to lipid metabolism, thus offering a therapeutic benefit in psoriasis management. This insight paves the way for lipid metabolism modulation to be considered as a novel and promising approach in psoriasis treatment strategies.

## Data Availability

The datasets presented in this study can be found in online repositories. The names of the repository/repositories and accession number(s) can be found in the article/Supplementary Material.
