# Supplementary material for: Network pharmacology and gut microbiota insights: unraveling Shenling Baizhu powder’s role in psoriasis treatment
Source: Front Pharmacol. 2024 Feb 14;15:1362161. doi: 10.3389/fphar.2024.1362161 (PMC10904012; doi:10.3389/fphar.2024.1362161)
Supplement: Supplementary file 1 [file Table1.DOCX]

Supplemental Table 1: Mass spectrum scanning parameter setting

| Mass spectrum scanning parameter | Set value |
| --- | --- |
| Scan mode | Full MS-ddms^2^ |
| Full MS scan range | 100 to 1500 *m*/*z* |
| Spectrum data type | Profile |
| Resolution | Full MS: 70,000 |
|  | MS/MS: 17,500 |
| AGC target | Full MS: 1e^6^ |
|  | MS/MS: 2e^5^ |
| Maximum IT | Full MS: 100 ms |
|  | MS/MS: 50 ms |
| Loop count | 3 |
| MSX count | 1 |
| Isolation width | 1.5 *m*/*z* |
| NCE (Stepped NCE) | 20, 40, 60 |
| Minimum AGC target | 8e^3^ |
| Intensity Threshold | 1.6e^5^ |
| Dynamic exclution | 5 s |
