# Supplementary material for: Network pharmacology and gut microbiota insights: unraveling Shenling Baizhu powder’s role in psoriasis treatment
Source: Front Pharmacol. 2024 Feb 14;15:1362161. doi: 10.3389/fphar.2024.1362161 (PMC10904012; doi:10.3389/fphar.2024.1362161)
Supplement: Supplementary file 2 [file Table2.DOCX]

Supplemental Table 2: The main components of SLBZP capsule

| NO. | Retention time (min) | Detection mode | Molecular weight | Molecular formula | deviation (ppm) | Composition identification | Source |
| --- | --- | --- | --- | --- | --- | --- | --- |
| 1 | 1.27 | Positive | 268.10358 | C8 H12 O3 N8 | -0.88 | Adenosine | Shan Yao |
| 2 | 4.51 | Negative | 165.05450 | C7 H7 O2 N3 | 0.76 | Unknown | Gan Cao |
| 3 | 9.85 | Negative | 417.11887 | C21 H21 O9 | -0.56 | Liquiritin | Gan Cao |
| 4 | 9.95 | Negative | 549.16125 | C26 H29 O13 | -0.21 | liquiritin apioside | Gan Cao |
| 5 | 10.84 | Positive | 625.32617 | C38 H45 O6 N2 | -1.67 | Neferine | Lian Zi |
| 6 | 11.09 | Negative | 447.09320 | C21 H19 O11 | -0.19 | Quercitrin | Sha Ren |
| 7 | 12.36 | Negative | 549.16132 | C26 H29 O13 | -0.08 | isoliquiritin apioside | Gan Cao |
| 8 | 12.64 | Negative | 417.11874 | C21 H21 O9 | -0.88 | Isoliquiritin | Gan Cao |
| 9 | 12.75 | Negative | 475.12421 | C23 H23 O11 | -0.79 | Ononin | Gan Cao |
| 10 | 12.94 | Negative | 255.06578 | C15 H11 O4 | -1.97 | Liquirtigenin | Gan Cao |
| 11 | 13.48 | Negative | 845.48944 | C43 H73 O16 | -0.71 | Ginsenoside Re | Ren Shen |
| 12 | 15.40 | Negative | 1237.54736 | C57 H89 O29 | -1.73 | Platycodin J/Platyconic acid A | Jie Geng |
| 13 | 15.86 | Negative | 1265.57715 | C59 H93 O29 | -2.88 | Platycodin A | Jie Geng |
| 14 | 16.15 | Negative | 983.44849 | C48 H71 O21 | -0.61 | licorice saponin A3 | Gan Cao |
| 15 | 16.45 | Negative | 845.48981 | C43 H73 O16 | -0.71 | Ginsenoside Rf | Ren Shen |
| 16 | 16.79 | Negative | 837.39050 | C29 H59 O19 N9 | -3.31 | licoricesaponin G2 isomer | Gan Cao |
| 17 | 16.85 | Negative | 255.06570 | C15 H11 O4 | -2.28 | Isoliquiritigenin | Gan Cao |
| 18 | 17.43 | Negative | 829.49438 | C43 H73 O15 | -1.04 | Ginsenoside Rg2 | Ren Shen |
| 19 | 17.61 | Negative | 1193.59521 | C57 H93 O26 | -0.71 | Malonyl- ginsenoside Rb1 | Ren Shen |
| 20 | 17.85 | Negative | 1123.58936 | C54 H91 O24 | -1.08 | Ginsenoside Rb2 | Ren Shen |
| 21 | 18.03 | Negative | 955.48975 | C48 H75 O19 | -1.10 | Ginsenoside Ro | Ren Shen |
| 22 | 18.11 | Negative | 837.39081 | C42 H61 O17 | -0.73 | licoricesaponin G2 | Gan Cao |
| 23 | 18.25 | Negative | 1123.58960 | C54 H91 O24 | -0.87 | Ginsenoside Rb2 or its isomer | Ren Shen |
| 24 | 18.39 | Negative | 1163.58484 | C56 H91 O25 | -0.56 | Malonyl- ginsenoside Rc/Malonyl- ginsenoside Rb2 | Ren Shen |
| 25 | 18.71 | Negative | 1195.60962 | C57 H95 O26 | -1.75 | Malonyl- ginsenoside Rb1 | Ren Shen |
| 26 | 19.10 | Negative | 821.39630 | C29 H59 O18 N9 | -2.50 | Glycyrrhizic acid | Gan Cao |
| 27 | 19.55 | Negative | 1165.59998 | C56 H93 O25 | -1.00 | Malonyl- ginsenoside Rc/Malonyl- ginsenoside Rb2 | Ren Shen |
| 28 | 20.06 | Negative | 821.39618 | C29 H59 O18 N9 | -2.65 | Glycyrrhizic acid isomer | Gan Cao |
| 29 | 20.62 | Negative | 1033.55847 | C51 H85 O21 | -0.40 | Malonyl- ginsenoside Rd | Ren Shen |
| 30 | 20.63 | Positive | 249.14821 | C15 H21 O3 | -1.25 | atractylenolide III | Bai Zhu |
| 31 | 20.81 | Negative | 367.11847 | C21 H19 O6 | -0.66 | glycycoumarin | Gan Cao |
| 32 | 21.15 | Negative | 823.41156 | C42 H63 O16 | -0.73 | uralsaponin C | Gan Cao |
| 33 | 21.87 | Negative | 353.10287 | C20 H17 O6 | -0.54 | Liconeolignan | Gan Cao |
| 34 | 22.33 | Negative | 381.13428 | C22 H21 O6 | -0.21 | licoricone | Gan Cao |
| 35 | 23.84 | Positive | 233.15327 | C15 H21 O2 | -1.44 | atractylenolide II | Bai Zhu |
| 36 | 23.90 | Negative | 351.08722 | C20 H15 O6 | -0.55 | semilicoisoflavone B | Gan Cao |
| 37 | 26.05 | Positive | 231.13774 | C15 H19 O2 | -0.94 | atractylenolide I | Bai Zhu |
| 38 | 26.38 | Negative | 483.31134 | C30 H43 O5 | -0.53 | Liquoric acid | Gan Cao |
| 39 | 26.38 | Negative | 483.31134 | C30 H43 O5 | -0.53 | Dehydrotumulosic acid | Fu Ling |
| 40 | 27.06 | Negative | 485.36337 | C31 H49 O4 | -0.54 | Tumulosic acid | Fu Ling |
| 41 | 28.23 | Negative | 481.33231 | C31 H45 O4 | -0.05 | Polyporenic acid C | Fu Ling |
| 42 | 32.55 | Negative | 527.3739 | C33 H51 O5 | -0.57 | Pachymic acid | Fu Ling |
